# Effect of Urea Spray on Boll Shell Insecticidal Protein Content in Bt Cotton

**DOI:** 10.3389/fpls.2021.623504

**Published:** 2021-05-11

**Authors:** Mingyuan Zhou, Zhenyu Liu, Linan Li, Yuan Chen, Xiang Zhang, Yuan Chen, Dehua Chen

**Affiliations:** Jiangsu Key Laboratory of Crop Genetics and Physiology, Co-innovation Center for Modern Production Technology of Grain Crops, Yangzhou University, Yangzhou, China

**Keywords:** Bt cotton, urea spray, boll shell, Bt protein, N metabolism, Cry1A

## Abstract

Reproductive organs of *Bacillus thuringiensis* transgenic cotton, which contribute to cotton final yield, have low insect resistant efficacy, so it is important to improve their insect resistance. This study was conducted to find out the impact of different urea spray doses on the expression of Cry1A protein in boll shell of Bt cotton (Sikang 1 and Sikang 3), and nitrogen metabolism in this process was also studied to uncover the physiological mechanism. The experiment with six urea doses was organized during peak boll stage in 2017 and 2018. The results showed that urea spray could significantly increase boll shell insecticidal protein contents in both cultivars, with the highest Bt protein content observed at 28–32 kg ha^−1^ urea dose. In addition, urea spray increased the contents of soluble protein and free amino acid and the activities of GS, GOGAT, GOT, and GPT, but decreased the activities of peptidase and protease in boll shell. Correlation analysis showed that the amount of boll shell Bt protein was positively correlated with levels of soluble protein and amino acid, and activities of GS, GOGAT, GOT, and GPT, but negatively correlated with peptidase and protease activities. Thus, this study demonstrated that higher protein synthesis ability and lower proteolysis ability were related to increased Bt protein content in urea-sprayed boll shell.

## Introduction

*Bacillus thuringiensis* transgenic cotton (Bt cotton) is the cotton cultivar with insect resistance, which is obtained by transferring Bt toxin gene from *B. thuringiensis* into cotton (Ma et al., 2020). In the whole process of growth and development of Bt cotton, all tissues and organs can express insecticidal protein, which can effectively reduce the harm of cotton bollworm and other related pests (Chen et al., 2005, 2018; Zhang et al., 2019; Zhou et al., 2019). In addition, the application of Bt cotton effectively reduced the use of pesticides (Fang et al., 2011; Qiao et al., 2017b) and thus decreased input and environmental pollution (Sun et al., 2016). Due to the huge economic and ecological benefits of Bt cotton all over the world since the mid-1990s (Frisvolda and Reeves, 2008; Qiao, 2015; Qiao et al., 2017a; Kouser et al., 2019), it is of great practical significance to ensure the efficient expression of Bt cotton insecticidal protein.

However, insecticidal performance was not consistent in different organs and growth stages of Bt cotton (Chen et al., 2004; Pettigrew and Adamczyk, 2006; Dong and Li, 2007; Carrière et al., 2019; Patricia et al., 2020), which was characterized by high insect resistance in leaves, but low insect resistance in reproductive organs of Bt cotton (Dong and Li, 2007; Glenn, 2011; Siddiqui et al., 2019). In the course of its growth and development, Bt cotton in boll stage has lower insecticidal efficacy than that in early growth stage (Adamczyk and Meredith, 2004). Therefore, it is of great significance to improve the insect resistance of Bt cotton reproductive organs in production. Previous studies had shown that the insecticidal efficacy was associated with the amount of Bt protein (Chen et al., 2004). The results of Dai’s study indicated that the application of nitrogen can increase the expression of insecticidal protein, so as to effectively alleviate the decrease of insect resistance of Bt cotton in late growth stage (Dai et al., 2012). Our previous studies discovered that the growth and physiological state of reproduction organs influenced the amount of Bt protein (Chen et al., 2017, 2018). In addition, the change of nitrogen metabolism was the major factor contributing to altered Bt protein expression (Chen et al., 2012, 2019). Urea, as a widely used nitrogen fertilizer, provides essential nutrients for plant growth and development. Nitrogen supplied by urea is the main source of nitrogen in plant nitrogen metabolism. Therefore, studying the relationship between urea application and insecticidal protein content of boll shell is quite important to improve the insecticidal ability of cotton bolls. The objective of this study was to ascertain the effects of spraying different urea doses on the expression of insecticidal protein, and nitrogen metabolism in this process was also studied to uncover the potential mechanism.

## Materials and Methods

### Plant Materials and Field Design

Field experiments were implemented on the farm of Yangzhou University, China during 2017 and 2018 cotton growing seasons. Sikang 1 (conventional) and Sikang 3 (hybrid), two Cry1A-expressing Bt cotton cultivars, were used in this study. Seeds were sown on April 15 (2017) and April 16 (2018) in a plastic-covered greenhouse. Seedlings were transplanted to field on May 13 (2017) and May 15 (2018), respectively. The planting densities of two cultivars were 37,500 plants ha^−1^ and 27,000 plants ha^−1^ for Sikang 1 and Sikang 3, respectively. The soil had a sandy loam texture, which contained 21.6 and 22.3 g kg^−1^ organic matter, and available N-P-K at 108.3 and 110.2, 24.2 and 25.3, and 82.1 and 81.3 mg kg^−1^ in 2017–2018, respectively. Fertilizer and plant growth retardant were applied as follows: P (300 kg ha^−1^ as superphosphate) and K (150 kg ha^−1^ as KCl) were top-dressed at seedling stage and early flowering, respectively. Urea was applied at seedling stage (163 kg ha^−1^), early flowering (130 kg ha^−1^), early boll developing stage (295 kg ha^−1^), and peak boll stage (65 kg ha^−1^). The DPC (C_7_H_16_ClN) was applied at peak square (15 g ha^−1^), early flower (30 g ha^−1^), peak flower (45 g·ha^−1^), and peak boll period (60 g ha^−1^).

In 2017 and 2018, a split plot design with three replications was applied. The main plot treatment was cultivars (Sikang 1 and Sikang 3), and the subplot treatment was six urea doses (0, 9, 18, 27, 36, and 45 kg ha^−1^ in 2017, and 0, 4.5, 13.5, 22.5, 31.5, and 40.5 kg ha^−1^ in 2018). Each plot consisted of six rows of cotton, and the plot dimension was 5.00 m × 4.15 m. We marked the flowers of the first position of the fourth to sixth fruiting branches of the plants at peak flowering stage and sprayed the plants 5 days before sampling. The urea was sprayed with pneumatic spray bottles (fan-type nozzle), and each subplot was sprayed with 1,000 ml of solution to ensure that the boll and its counterpart leaf were moist.

### Preparation of Plant Material

In the 2017 and 2018 urea application experiment, the amount of boll shell insecticidal protein at 15, 20, and 25 days after flowering was measured during the boll stage. At each sampling time, for each plot, five bolls were harvested from the first position of the fourth to sixth fruiting branches of the plants for further analysis. The bolls were frozen with liquid nitrogen, stored in ultra-low-temperature freezer, and used for Bt protein content, nitrogen metabolic chemicals, and enzyme activity measurement.

### Physiological Measurements

#### The Bt Protein Content

The insecticidal protein contents of cotton boll shell were determined by ELISA (Dong et al., 2000). Three samples of boll shell tissue extracts were prepared by homogenizing the frozen tissue in 2 ml of extraction buffer (NaCl 8.0 g, KH_2_PO_4_ 0.2 g, and Na_2_HPO_4_ 2.96 g dissolved in 1,000 ml of distilled water; 1 ml Tween). The centrifuge tube was shaken with hand, stored at 4°C for 4 h, and then centrifuged at 1000 × *g* for 15 min. The supernatant is the sample to be tested.

Quantification of the boll shell insecticidal protein was conducted using a commercially available kit (Scientific Service, Inc., China Agriculture University) according to Chen et al. (2019). Microtitration plates were coated with 100 μl of buffer solution (Na_2_CO_3_ 1.5 g, NaHCO_3_ 2.93 g dissolved in 1,000 ml of distilled water, pH 9.6; 1 ml anti-rabbit immunoglobulin) and incubated at 37°C for 4 h. The 50-μl antibodies, standard CryIA insecticidal proteins, and samples were added to each well and incubated for further 30 min at 37°C. Then, 100 μl of horseradish peroxidase-labeled goat anti-rabbit immunoglobulin was added to each well and incubated for 30 min at 37°C. Finally, 100 μl of buffered enzyme substrate (o-Penylenediamine 0.2 g, C_6_H_8_O_7_·H_2_O 0.51 g, and Na_2_HPO_4_·12H_2_O 1.843 g dissolved in 100 ml distilled water, pH 5.0; 0.1 ml Tween; 40 μl 30% H_2_O_2_) was added, and the enzyme reaction was carried out in the dark at 37°C for 15 min and then terminated using 50 μl of 2 mol·L^−1^ H_2_SO_4_. The absorbance was recorded at 490 nm.

#### Soluble Protein and Amino Acid Content

The boll shell samples from different treatments were used for soluble protein content analysis. The samples were homogenized at 4°C in 1.5 ml of cold water and centrifuged at 7100 × *g* for 10 min. The supernatant is the sample to be tested. The total soluble protein content was determined by the Coomassie Blue dye-binding assay of Zou (2000).

Three samples of boll shell tissue extracts were prepared by homogenizing the frozen tissue in 1 ml of extracting solution (10% CH_3_COOH). The samples were centrifuged at 1000 × *g* for 5 min. The supernatant was used for the analysis of amino acid concentration. The total free amino acid content was determined by ninhydrin assay (Zou, 2000).

#### Glutamine Synthetase and Glutamate Synthetase Assay

The boll shell samples were homogenized in 1.5 ml of 0.1 mol L^−1^ Tris-HCl extraction buffer (12.114 g Tris, 0.095 g MgCl_2_, 0.292 g EDTA, and 0.704 ml 2-Hydroxy-1-ethanethiol dissolved in 1,000 ml of distilled water, pH adjusted to 7.6 with 1 mol·L^−1^ HCl), and the homogenate was centrifuged at 18,500 × *g* for 25 min at 4°C. The supernatant was analyzed for GS and GOGAT activity. The experiment was conducted according to Sun et al. (2009).

A mixture of 0.3 ml of 0.25 mol L^−1^ buffer solution (imidazole 17.02 g dissolved in 1,000 ml of distilled water, pH 7.0), 0.2 ml of 0.3 mol L^−1^ sodium glutamate, 0.2 ml of 30 mmol L^−1^ ATP-Na, 0.1 ml of 0.5 mol L^−1^ MgSO_4_, 0.1 ml of 0.8 mol L^−1^ hydroxylamine hydrochloride, and 0.6 ml of the enzyme preparation was incubated at 25°C for 1 h and then terminated using 0.4 ml of stop solution (TCA 33.18 g, FeCl_3_ 101.02 g, and 5 ml of HCl dissolved in 1000 ml of distilled water). The reaction mixture was centrifuged at 5000 × *g* for 15 min. The absorbance of the supernatant was recorded at 540 nm. The GS activity was expressed by the change of absorbance.

Microtitration plates were coated with 40 μl of 20 mmol·L^−1^ l-glutamine, 50 μl of 20 mmol·L^−1^ α-ketoglutaric, 10 μl of 10 mmol·L^−1^ KCl, 20 μl of 3 mmol·L^−1^ NADH, and 30 μl of the enzyme preparation. The reaction mixture was recorded at 340 nm per 10 s for 5 min. The GOGAT activity was expressed by the amount of NADH reduction.

#### Glutamate Oxaloacetate Transaminase and Glutamic-Pyruvic Transaminase Assay

The boll shell samples were homogenized in 2 ml of 0.05 mol·L^−1^ Tris-HCl extraction buffer (6.05 g Tris and 22.1 ml 2 mol·L^−1^ HCl dissolved in 1,000 ml of distilled water, pH 7.2), and the homogenate was centrifuged at 20,000 × *g* for 20 min at 4°C. The supernatant was analyzed for GOT and GPT activity. The experiment was conducted according to Wu et al. (1998).

A mixture of 0.5 ml of substrate solution (200 mmol L^−1^ DL-aspartic acid, 2 mmol L^−1^ α-ketoglutaric, pH 7.4) and 0.1 ml of the enzyme preparation was incubated at 37°C for 1 h and then terminated using 0.5 ml of 1 mmol L^−1^ 2,4-dinitrophenylhydrazine. The reaction mixture was incubated for further 20 min at 37°C and then 5.0 ml of 4 mol L^−1^ NaOH was added. The absorbance was recorded at 500 nm. The GOT activity, in terms of pyruvate production, was calculated from authentic pyruvate standards run simultaneously.

A mixture of 0.5 ml of substrate solution (200 mmol L^−1^ alanine, 2 mmol L^−1^ α-ketoglutaric, pH 7.4) and 0.1 ml of the enzyme preparation was incubated at 37°C for 30 min and then terminated using 0.5 ml of 1 mmol·L^−1^ 2,4-dinitrophenylhydrazine. The reaction mixture was incubated for further 20 min at 37°C and then 5.0 ml of 4 mol·L^−1^ NaOH was added. The absorbance was recorded at 500 nm. The GPT activity, in terms of pyruvate production, was calculated from authentic pyruvate standards run simultaneously.

#### Assay of Protease and Peptidase Activity

The boll shell samples were homogenized in 1.5 ml of water and the homogenate was shaken for 30 min at a 40°C water bath. Then, the homogenate was centrifuged at 14,200 × *g* for 10 min at 10°C. Protease activity was determined by the Folin-Ciocslteu assay of Dong et al. (2000). A mixture of 0.25 ml of substrate solution (2% casein, pH 7.0) and 0.25 ml of the enzyme preparation was incubated at 40°C for 10 min and then terminated using 0.5 ml of 0.4 mol·L^−1^ TCA (trichloroacetic acid). The reaction mixture was incubated for further 10 min at 40°C and then centrifuged at 1000 × *g* for 2 min. Finally, 0.5 ml of supernatant, 2.5 ml of 0.4 mol·L^−1^ Na_2_CO_3_, and 0.5 ml of 25% Folin-Ciocslteu were added to the centrifuge tubes and incubated for 20 min at 40°C. The absorbance was recorded at 660 nm. The protease activity, in terms of tyrosine production, was calculated from authentic tyrosine standards run simultaneously.

The boll shell samples were homogenized at 4°C in 1.5 ml of 5 mmol·L^−1^ Hepes extraction buffer (2.383 g Hepes, 0.7445 g EDTA-Na_2_, 0.617 g DTT, and 20 g PVP dissolved in 2000 ml of distilled water, pH 8.0) and then centrifuged at 15,000 × *g* for 20 min at 4°C. The supernatant was used to estimate the peptidase activity. The analysis was conducted according to Sun et al. (2009). A mixture of 0.1 ml of substrate solution (2% bovine hemoglobin, pH 5.2), 0.7 ml of 200 mmol·L^−1^ citric acid buffer, and 0.2 ml of the enzyme preparation was incubated at 38°C for 1 h and then terminated using 0.8 ml of 12% TCA. The reaction mixture was incubated for further 30 min at 4°C and then centrifuged at 4000 × *g* for 2 min. The supernatant was used for the analysis of the amino acid content, which was determined by ninhydrin assay (Zou, 2000). The absorbance was recorded at 570 nm.

### Statistics Analysis

Data were analyzed by Proc ANOVA in SPSS. The significance of difference among different treatments were tested by LSD at *p* ≤ 0.05. The Pearson correlation coefficient was used to calculate the correlation.

## Results

### Boll Shell Bt Protein Content Under Different Urea Doses

**Figure 1:**
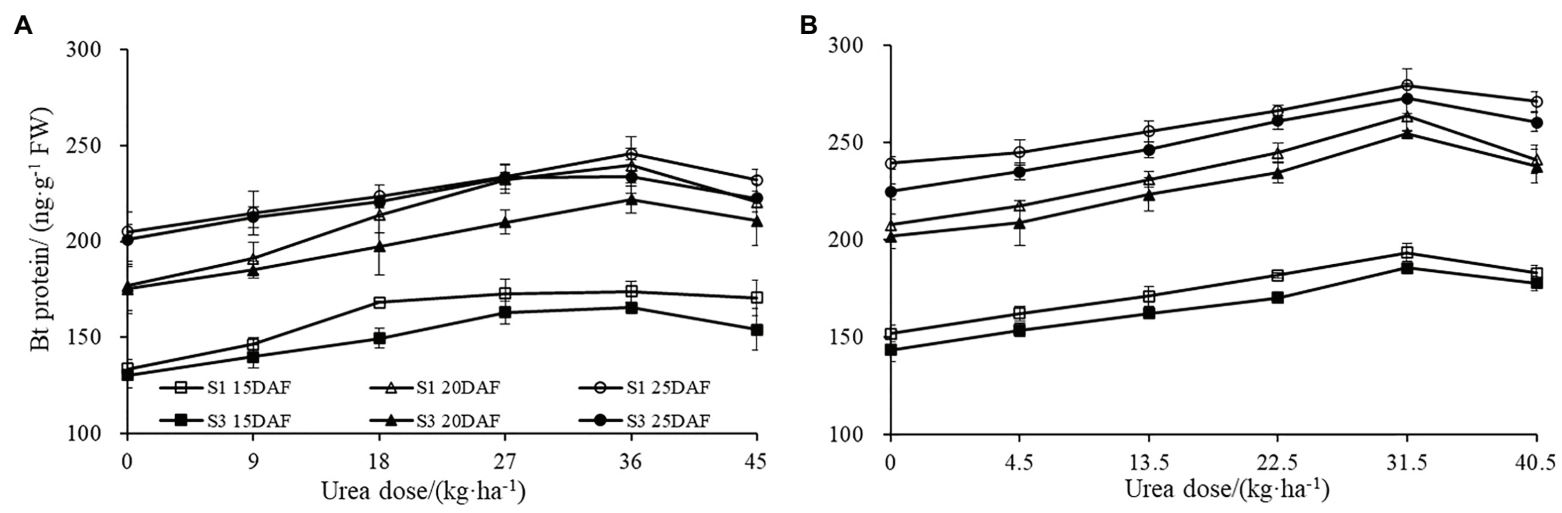
The boll shell Bt protein content under different urea doses in 2017 **(A)** and 2018 **(B)**. FW represents the fresh weight of the sample, DAF represents the abbreviation of “days after flowering,” and S1 and S3 represent the Sikang 1 and Sikang 3, respectively.

Similar trends were observed for Bt protein content under different urea doses during 2017 and 2018 growth seasons (Figure 1). Boll shell Bt protein content increased as urea doses increased, and decreased as urea dose exceeded 31.5 kg ha^−1^ (2018) and 36 kg ha^−1^ (2017). The different concentration of urea had a significant effect on the amount of boll shell insecticidal protein. A greater increase of Bt protein content in the boll shell was detected under 36 kg·ha^−1^ urea, with an increase of 35.48% in Sikang 1 and 26.56% in Sikang 3 compared to untreated control at 20DAF in 2017. Similar trends were recorded in 2018. Enhanced boll shell Bt protein contents were also observed with developmental process for both cultivars in 2017 and 2018. Greater increases were observed from 15 to 20 days than that from 20 to 25 days. The amount of boll shell Bt protein content was increased by 37.81% from 15 to 20 DAF, compared to 2.55% from 20 to 25 DAF under 36 kg·ha^−1^ urea for Sikang 1 in 2017. Similar trends were observed for cultivar Sikang 3.

### Boll Shell Nitrogen Metabolism Under Different Urea Doses

**Figure 2:**
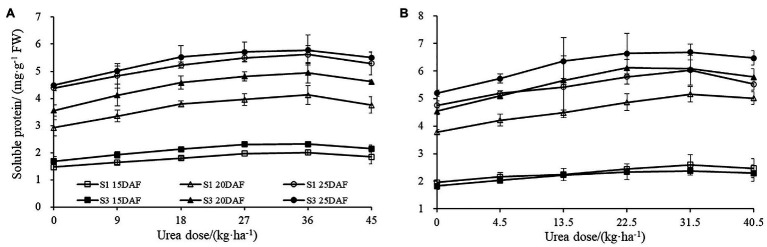
The boll shell soluble protein content under different urea doses in 2017 **(A)** and 2018 **(B)**. FW represents the fresh weight of the sample, DAF represents the abbreviation of “days after flowering,” and S1 and S3 represent the Sikang 1 and Sikang 3, respectively.

The soluble protein content of the boll shell increased with developmental process for both cultivars in 2017 and 2018 (Figure 2). The enhanced extents of boll shell soluble protein level varied under different urea doses. Greater increases of soluble protein contents were observed under high urea doses, and less increases were detected under low urea doses. Greater increase of protein content in boll shell was detected under 36 kg ha^−1^ urea, with an increase of 41.44% in Sikang 1 and 39.16% in Sikang 3 compared to untreated control at 20 DAF in 2017.

**Figure 3:**
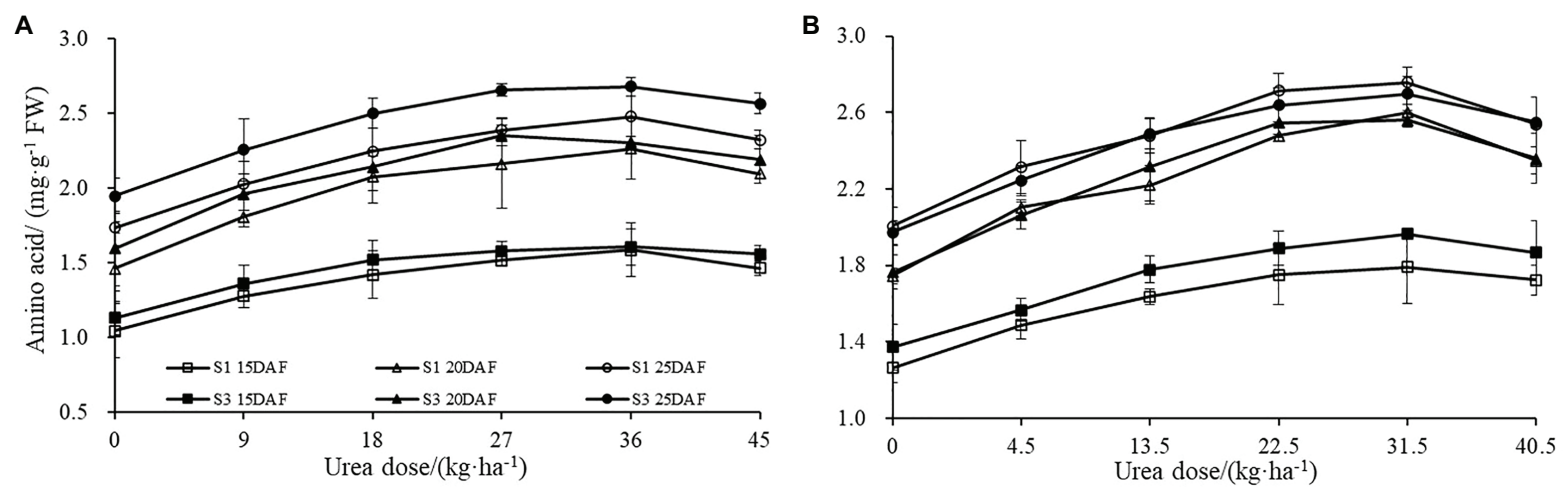
The boll shell amino acid content under different urea doses in 2017 **(A)** and 2018 **(B)**. FW represents the fresh weight of the sample, DAF represents the abbreviation of “days after flowering,” and S1 and S3 represent the Sikang 1 and Sikang 3, respectively.

Similar effects were also observed for boll shell amino acid content (Figure 3). Boll shell amino acid content was enhanced when urea doses increased from 0 to 36 kg ha^−1^, and decreased as urea dose exceeded 36 kg·ha^−1^ for both cultivars. Enhanced boll shell amino acid contents were also observed with developmental process for both cultivars in 2017 and 2018. Greater increases were observed from 15 to 20 days than that from 20 to 25 days. In 2017, the amount of boll shell amino acid content was increased by 42.66% from 15 to 20 DAF, compared to 9.55% from 20 to 25 DAF under 36 kg·ha^−1^ urea for Sikang 1. Similar trends were observed for cultivar Sikang 3.

**Figure 4:**
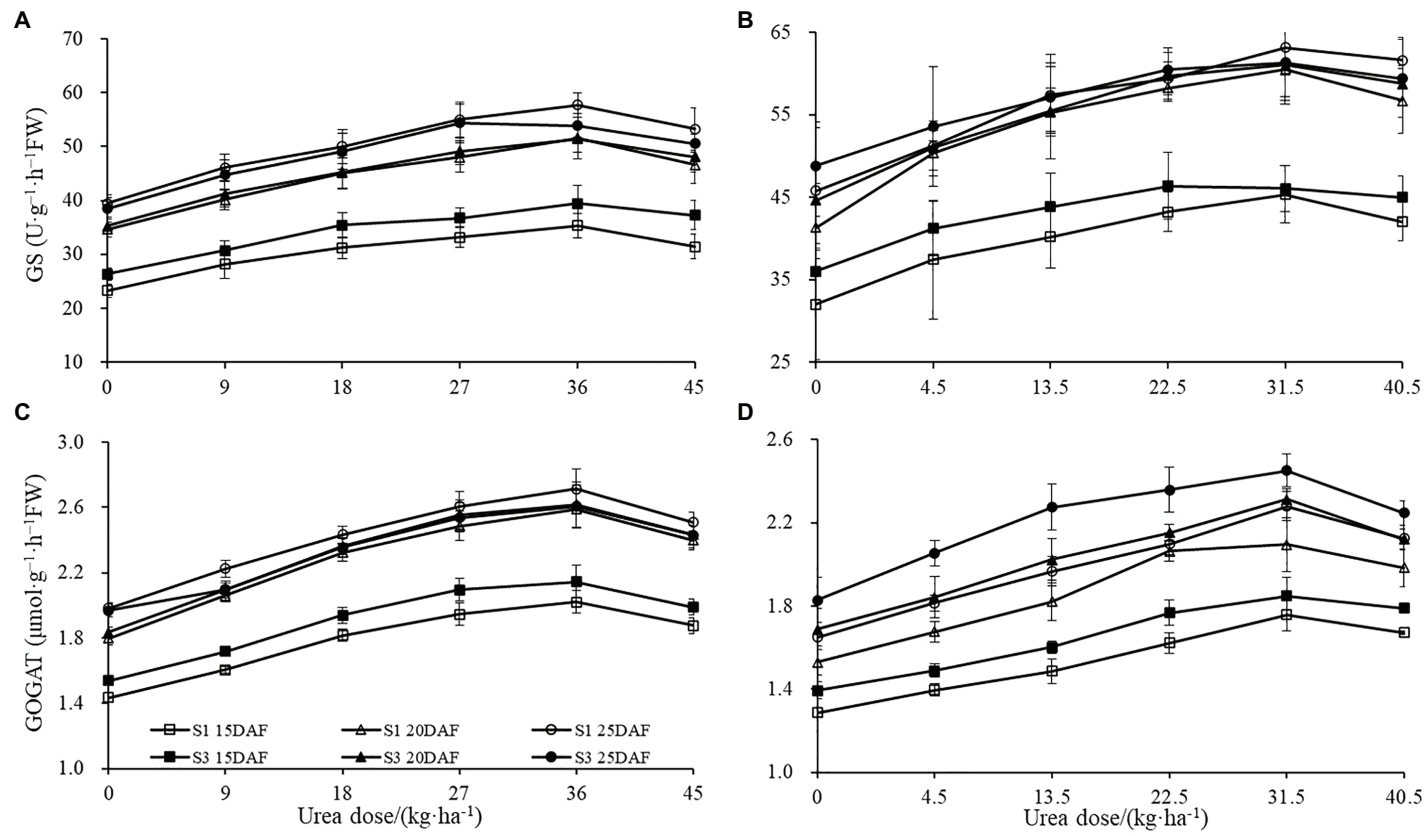
The boll shell GS and GOGAT activity under different urea doses in 2017 (**A**,**C**) and 2018 (**B**,**D**). GS represents the abbreviation of “glutamine synthetase,” GOGAT represents the abbreviation of “glutamate synthetase,” FW represents the fresh weight of sample, DAF represents the abbreviation of “days after flowering,” and S1 and S3 represent the Sikang 1 and Sikang 3, respectively.

The different concentrations of urea had a significant effect on the boll shell GS and GOGAT activities (Figure 4). Greater increase of GS and GOGAT activities in the boll shell was detected under 36 kg ha^−1^ urea, with an increase of 49.10 and 44.10% in Sikang 1 and 45.83 and 42.42% in Sikang 3 compared to untreated control at 20 DAF in 2017. Enhanced boll shell GS and GOGAT activities were also observed with developmental process for both cultivars in 2017 and 2018. Greater increases were observed from 15 to 20 days than that from 20 to 25 days. In 2017, the boll shell GS and GOGAT activities were increased by 46.01 and 28.12% from 15 to 20 DAF, compared to 11.90 and 4.78% from 20 to 25 DAF under 36 kg ha^−1^ urea for Sikang 1. Similar trends were seen for cultivar Sikang 3.

**Figure 5:**
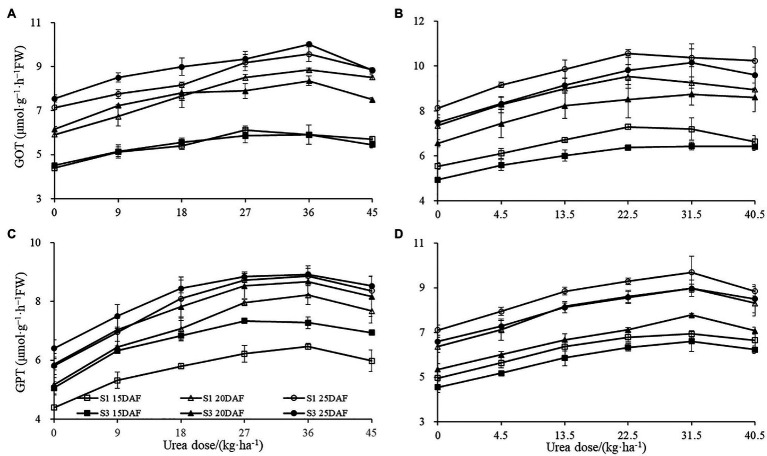
The boll shell GOT and GPT activity under different urea doses in 2017 (**A**,**C**) and 2018 (**B**,**D**). GOT represents the abbreviation of “glutamate oxaloacetate transaminase,” GPT represents the abbreviation of “glutamic-pyruvic transaminase,” FW represents the fresh weight of the sample, DAF represents the abbreviation of “days after flowering,” and S1 and S3 represent the Sikang 1 and Sikang 3, respectively.

The different concentrations of urea had a significant effect on boll shell GOT and GPT activities in 2017 and 2018 (Figure 5). However, the enhanced extents of boll shell GOT and GPT activities varied under different urea doses. Greater increase of GOT and GPT activities in boll shell was detected under 36 kg ha^−1^ urea, with an increase of 49.40 and 58.76% in Sikang 1 and 35.62 and 48.11% in Sikang 3, respectively, compared to untreated control at 20 DAF in 2017. Enhanced boll shell GOT and GPT activities were also observed with developmental process for both cultivars in 2017 and 2018.

**Figure 6:**
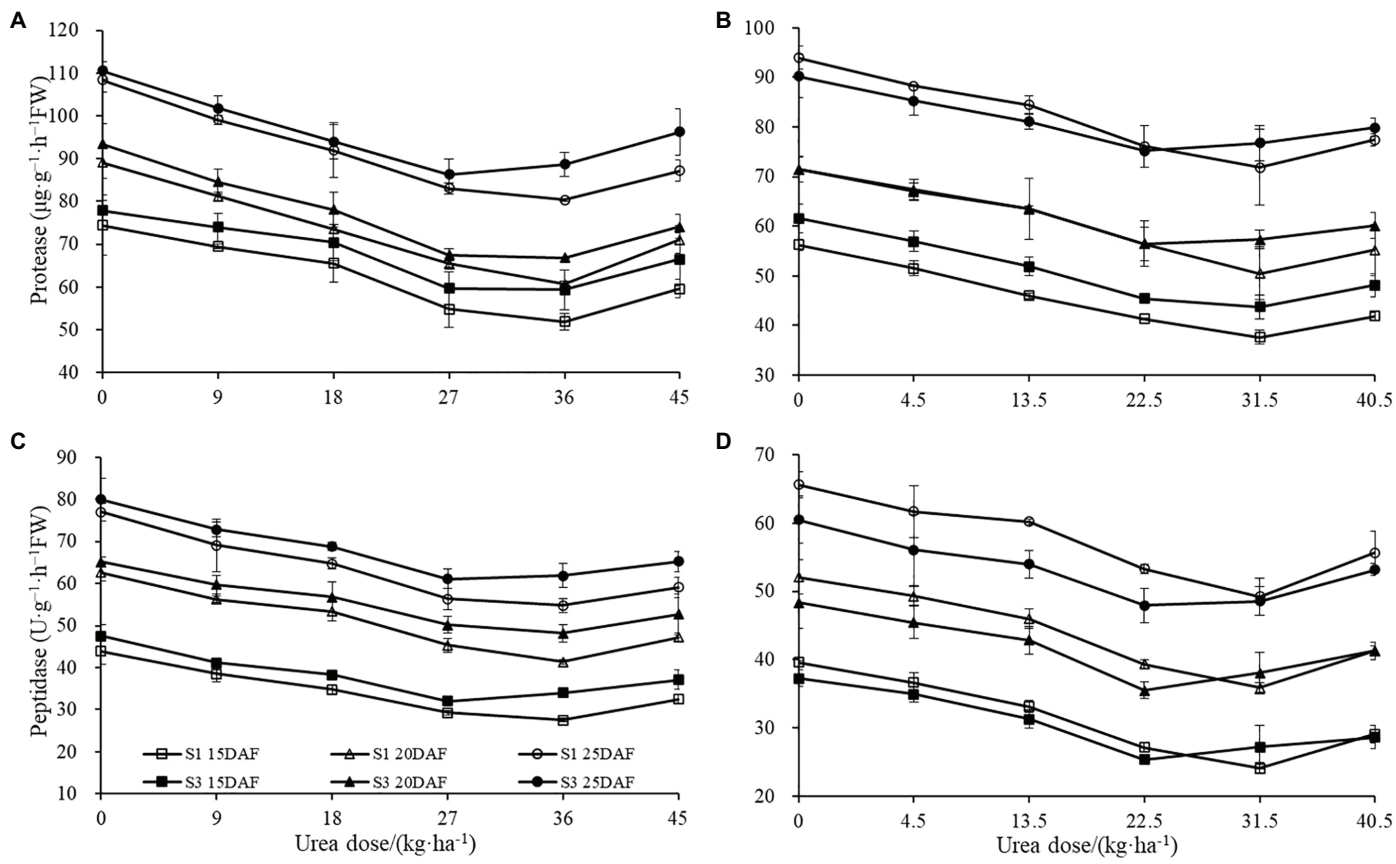
The boll shell protease and peptidase activities under different urea doses in 2017 (**A**,**C**) and 2018 (**B**,**D**). FW represents the fresh weight of the sample, DAF represents the abbreviation of “days after flowering,” and S1 and S3 represent the Sikang 1 and Sikang 3, respectively.

The different concentration of urea had a significant effect on boll shell protease and peptidase activities (Figure 6). Boll shell protease and peptidase activities decreased when urea doses increased from 0 to 36 kg ha^−1^ and enhanced as urea dose exceeded 36 kg ha^−1^ for both cultivars. Greater decrease of protease and peptidase activities in boll shell was detected under 36 kg ha^−1^ urea, with a decrease of 31.91 and 33.95% in Sikang 1 and 28.41 and 26.00% in Sikang 3, respectively, compared to untreated control at 20 DAF in 2017. In contrast, the boll shell protease and peptidase activities enhanced with growth process for both cultivars.

### Correlation of Bt Protein Content With Related Parameters in Nitrogen Metabolism

Boll shell soluble protein content, GPT, GS, and GOGAT activities were positively correlated with Bt protein content under different urea spraying treatments in 2017 and 2018 (Table 1), while a significantly negative correlation was observed between Bt protein content with activities of protease and peptidase. A stronger correlation between amino acid and Bt protein content was observed in 2017 than in 2018.

**Table 1:** Analysis of relation between insecticidal protein and enzymatic activities in Bt cotton.

Years	Cultivar	Soluble protein	Amino acid	GS activity	GOGAT activity	GOT activity	GPT activity	Protease activity	Peptidase activity
2017	Sikang 1	0.9608^**^	0.9731^**^	0.9544^**^	0.9818^**^	0.9461^**^	0.9654^**^	−0.9022^*^	−0.9485^**^
	Sikang 3	0.9500^**^	0.9174^**^	0.9815^**^	0.9709^**^	0.7786	0.9599^**^	−0.9667^**^	−0.9842^**^
2018	Sikang 1	0.9347^**^	0.9154^*^	0.9663^**^	0.9885^**^	0.8971^*^	0.9269^**^	−0.9836^**^	−0.9629^**^
	Sikang 3	0.9082^**^	0.9131^*^	0.9377^**^	0.9852^**^	0.9194^**^	0.9840^**^	−0.9190^**^	−0.8492^*^

GS, glutamine synthetase; GOGAT, glutamate synthetase; GOT, glutamate oxaloacetate transaminase; GPT, glutamic-pyruvic transaminase.

* 5% significant level.

** 1% extremely significant level.

## Discussion

### Proper Urea Spray Dose Increased Boll Shell Insecticidal Protein Content in Bt Cotton

**Figure 7:**
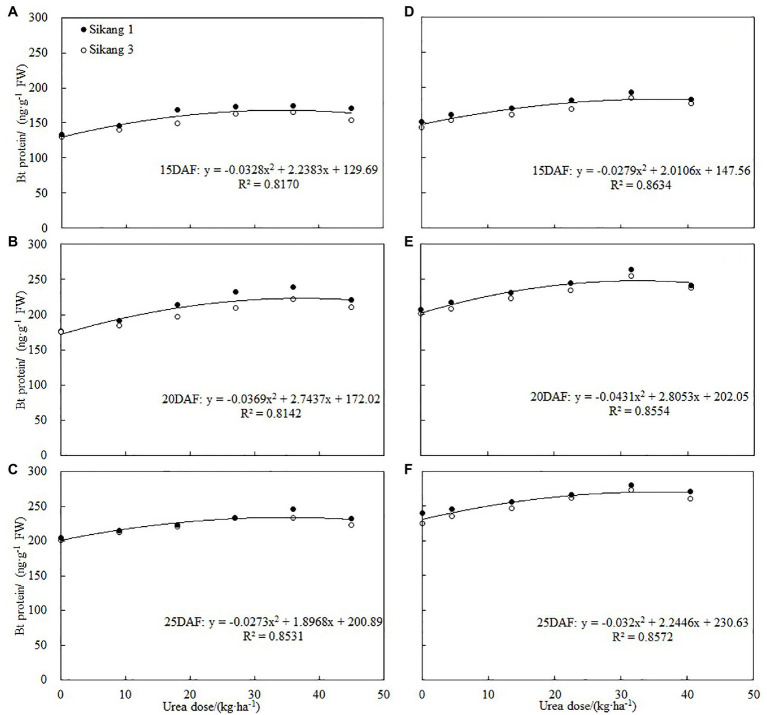
Correlations between boll shell Bt protein content and urea dose at 15DAF (**A**,**D**), 20DAF (**B**,**E**), and 25DAF (**C**,**F**) in 2017 and 2018, respectively. FW represents the fresh weight of the sample, and DAF represents the abbreviation of “days after flowering.”

Many studies showed that boll shell and seed in Bt cotton exhibited lower insect resistance compared to nutritive organ (Wang et al., 2009; Glenn, 2011; Chen et al., 2018; Zhang et al., 2019). Boll shell is the first defense for boll resistance, and bollworms attack it first in order to enter cotton bolls. Therefore, it is of great significance to improve the insect resistance of Bt cotton by increasing the content of insecticidal protein in boll shell. Previous studies have shown that nitrogen fertilizer could affect the expression of Bt cotton insecticidal protein, and the expression of Bt protein is closely related to nitrogen metabolism (Chen et al., 2005, 2013, 2017; Dong and Li, 2007). Some studies have shown that increasing nitrogen fertilizer or spraying amino acid can increase the expression of insecticidal protein in reproductive organs such as squares, flowers, and bolls (Pettigrew and Adamczyk, 2006; Abidallha et al., 2017a,b; Chen et al., 2018; Zhou et al., 2019). The results of our study further showed that spraying urea could significantly increase the expression of insecticidal protein in boll shell, and as urea concentration enhanced, the expression of insecticidal protein increased first and then decreased, with the highest level recorded under 36 kg·ha^−1^ urea. Correlation analysis detected that there was a quadratic equation relationship between Bt protein and urea dose, which indicated that the content of Bt protein would begin to decline when the amount of urea reached a certain level (Figure 7). According to these studies, the application of nitrogen fertilizer and spraying amino acid and urea all resulted in enhanced expression of insecticidal protein in Bt cotton, but spraying urea is time-saving, labor-saving, and more economic, which is worthy of further study and technology promotion.

### Altered Boll Shell Nitrogen Metabolism Affected Bt Protein Content Under Urea Spray

Previous research has reported the factors influencing the expression of Bt insecticidal protein and its corresponding mechanism (Chen et al., 2005, 2017; Wang et al., 2020). Some studies have suggested that tannins produced by cotton plants could bind to insecticidal proteins, which changed the structure of insecticidal protein and inactivated it (Ma et al., 2012); others have suggested that the decreased expression of insecticidal proteins was caused by inactivation of insecticidal protein by methylation promoter of Bt gene (Yunus et al., 2019; Patricia et al., 2020); it has also been suggested that changes in the ability of protein synthesis and decomposition can alter the expression of insecticidal proteins (Chen et al., 2004, 2005; Dong and Li, 2007). Chen et al. (2017) showed that elevated nitrogen metabolism was beneficial for square development and insect resistance. The result of Chen’s study showed that the activities of GS, GOGAT, GOT, GPT, protease, and peptidase in Bt cotton leaves were closely related to the expression of insecticidal protein. Our present results showed that spraying urea increased the activities of GS, GOGAT, GOT, and GPT, but decreased the activities of peptidase and protease in boll shell. Correlation analysis showed that boll shell Bt protein content was positively correlated with activities of GS, GOGAT, GOT, GPT, and negatively correlated with activities of peptidase and protease, indicating that the enhanced boll shell Bt protein content was the result of elevated protein synthesis ability and reduced protein decomposition ability. Further analysis showed that the content of Bt protein in boll shell in Sikang 1 was higher than that in Sikang 3, the activities of GS and GOGAT in Sikang 1 increased more than that in Sikang 3, while the protease and peptidase in Sikang 1 decreased more than that in Sikang 3. This result further confirmed that the change of nitrogen metabolism affected the content of insecticidal protein in boll shell.

## Conclusion

This study indicated that urea spraying increased the contents of soluble protein and free amino acid and the activities of GS, GOGAT, GOT, and GPT, but decreased the activities of peptidase and protease in boll shell of Bt cotton. Our results suggested that higher protein synthesis ability and lower proteolysis ability was significantly associated with Bt protein content in boll shell. The beneficial effect of urea spraying on boll shell Bt protein concentration suggested that we could improve the insect resistance of Bt cotton through a series of cultivation practices.

## Data Availability Statement

MZ: methodology, formal analysis, and writing - original draft preparation. MZ, ZL, and LL: investigation. YC (6th author) and DC: resources, supervision, and project administration. YC (4th author) and XZ: writing-review and editing. All authors contributed to the article and approved the submitted version.

## Author Contributions

MZ: methodology, formal analysis, and writing—original draft preparation. MZ, ZL, and LL: investigation. YC (4th author) and DC: resources, supervision, and project administration. YC (6th author) and XZ: writing—review and editing. All authors contributed to the article and approved the submitted version.

### Conflict of Interest

The authors declare that the research was conducted in the absence of any commercial or financial relationships that could be construed as a potential conflict of interest.

## Abbreviations:

DAF: Days after flowering
DPC: 1,1-Dimethyl piperidylium chloride
FW: Fresh weight
GOGAT: Glutamate synthetase
GOT: Glutamate oxaloacetate transaminase
GPT: Glutamic-pyruvic transaminase
GS: Glutamine synthetase.
